# Graph Theoretical Analysis of Semantic Fluency in Patients with Parkinson's Disease

**DOI:** 10.1155/2022/6935263

**Published:** 2022-04-23

**Authors:** Guanyu Zhang, Jinghong Ma, Piu Chan, Zheng Ye

**Affiliations:** ^1^China Institute of Sport Science, Beijing, China; ^2^Institute of Psychology, Chinese Academy of Sciences, Beijing, China; ^3^Department of Neurology, Xuanwu Hospital of Capital Medical University, Beijing, China; ^4^Department of Neurology and Neurobiology, National Clinical Research Center for Geriatric Disorders, Xuanwu Hospital of Capital Medical University, Beijing, China; ^5^Institute of Neuroscience, Center for Excellence in Brain Science and Intelligence Technology, Chinese Academy of Sciences, Shanghai, China

## Abstract

Semantic fluency is the ability to name items from a given category within a limited time, which relies on semantic memory, working memory, and executive function. Semantic disfluency is a common problem in Parkinson's disease (PD) and Alzheimer's disease (AD). We demonstrated a graph theoretical analysis of semantic fluency in patients with PD (*N* = 86), patients with AD (*N* = 40), and healthy controls (HC, *N* = 88). All participants completed a standard animal fluency test. Their verbal responses were recorded, transcripted, and transformed into directed speech graphs. Patients with PD generated fewer correct words than HC and more correct words than patients with AD. Patients with PD showed higher density, shorter diameter, and shorter average shortest path length than HC, but lower density, longer diameter, and longer average shortest path length than patients with AD. It suggests that patients with PD produced relatively smaller and denser speech graphs. Moreover, in PD, the densities of speech graphs correlated with the severity of non-motor symptoms, but not the severity of motor symptoms. The graph theoretical analysis revealed new features of semantic disfluency in patients with PD.

## 1. Introduction

Semantic fluency is the ability to name items from a given category (e.g., animals) during a given time interval, usually one minute (semantic fluency test). This task is significantly influenced by semantic memory (e.g., semantic representations to be organized), working memory (e.g., keeping the search for new satisfying words), and executive function (e.g., the ability to select and retrieve correct words and inhibit those that are not inherent with the specific category) domains. Semantic disfluency is a common problem in Parkinson's disease (PD) and Alzheimer's disease (AD). Patients with PD or AD generate fewer correct words than healthy adults in the semantic fluency test [1–4].

Different approaches have been developed to quantify verbal responses in semantic fluency tests. Troyer and colleagues [5] proposed a method to segment the verbal response into clusters according to the semantic relatedness between words. For example, a participant may begin with farm animals (e.g., ox, horse, and donkey) and then switch to forest animals (e.g., wolf, bear, and fox). This method generates two primary parameters: the mean cluster size, which is the average number of sequential words from the same subcategory, and the number of switches between subcategories. It is assumed that the mean cluster size reflects semantic storage in the temporal lobe and the number of switches reflects executive functions in the frontal lobe. PD patients with dementia or mild cognitive impairment often switch less than healthy adults but they do not necessarily produce smaller clusters [6, 7]. In contrast, patients with AD switch less and produce smaller clusters than healthy adults [6].

The Troyer method relies heavily on experimenters' subjective judgment of semantic relatedness and cluster segmentation. Farzanfar et al. compared an automated computational assessment with the traditional experimenter-based assessment of semantic fluency data from patients with PD [8]. In the computational assessment, each word was represented as a vector in a semantic space derived from corpora. Semantic relatedness between a given pair of words was defined as the cosine of the angle between the corresponding vectors (range from -1 [low relatedness] to 1 [high relatedness]). Sequential words with a semantic relatedness value higher than a predetermined threshold (0.5-0.6) were members of the same cluster. A semantic relatedness value below the threshold indicated a switch between clusters. The computational assessment was inconsistent with the experimenter-based assessment in detecting clusters: the correlation between the two assessments varied as a function of the threshold. In the experimenter-based assessment, the estimation of cluster and switch might be biased by the experimenter's semantic knowledge.

An objective method is based on graph theory. Graph theory has been used to reveal topological changes in brain networks in various brain disorders [9–11]. Recently, Bertola and colleagues [12] used graph theory to analyze semantic fluency data of patients with AD or mild cognitive impairment. They found that speech graphs of semantic fluency become smaller and denser as general cognition decreases. In another study, Mota and colleagues [13] used a graph theory to analyze dream reports and found that speech graphs of patients with schizophrenia were less connected than those of healthy adults. The individual patients' connectivity within speech graphs correlated with their severity of negative and cognitive symptoms. As a sensitive measurement, we hypothesize that the graph theoretical analysis can extract more semantic features, which potentially contributes to the identification of mild cognitive impairment in PD from healthy adults or AD.

**Figure 1:**
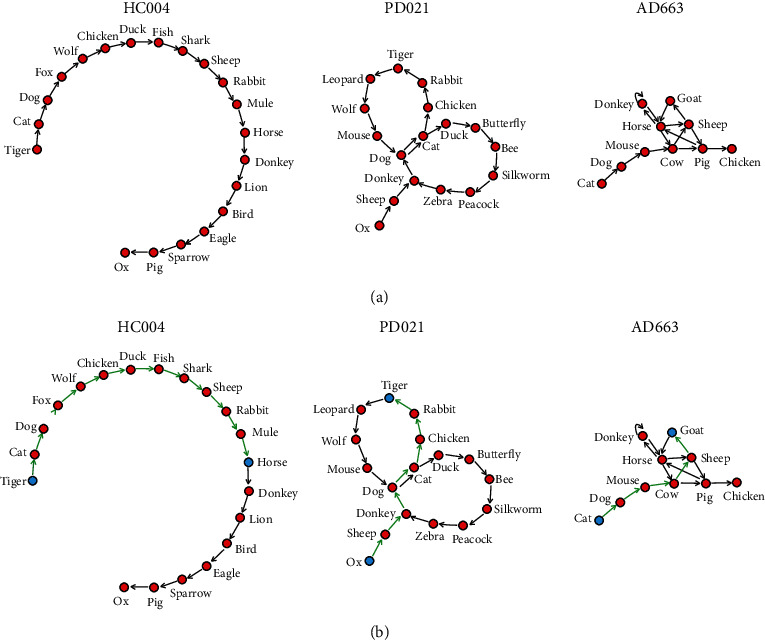
(a) Directed speech graphs of three representative participants. HC004, a healthy control subject; PD021, a patient with Parkinson's disease; AD663, a patient with Alzheimer's disease. (b) Graph geodesic as the shortest path (green) between two nodes (blue) in the three participants.

In this study, we revisit the semantic disfluency of patients with PD, comparing speech graphs of patients with PD with those of healthy adults and patients with AD. All participants completed a standard animal fluency test. We transformed participants' verbal responses into directed speech graphs, with each node representing a correct word and each arc representing a temporal link between sequential words (Figure 1). First, we wanted to detect group differences in the number of correct words, repetitions, incorrect words, metalinguistic reference, and metacognitive reference (standard analysis). Second, we sought group differences in global characteristics of speech graphs, including density, diameter, and average shortest path (graph theoretical analysis). Third, in PD, we explored whether the speech graph parameters correlated with clinical features such as the severity of motor or non-motor symptoms.

## 2. Materials and Methods

This study was approved by the ethics committee of the Xuanwu Hospital according to the Declaration of Helsinki. Each participant signed a written informed consent before participating in this study.

### 2.1. PD Patients and Clinical Assessments

We included 86 patients with idiopathic PD (Movement Disorder Society Clinical Diagnostic Criteria for Parkinson's Disease [14]) at the Xuanwu Hospital Research and Clinical Center for Parkinson's disease between 2017 and 2019. Inclusion criteria were (1) Hoehn and Yahr Stages 1 to 2; (2) age 40 to 80 years; (3) education ≥6 years; and (4) Mandarin Chinese speaking. Exclusion criteria were (1) a history of epilepsy, stroke, or brain injury; (2) alcohol or drug abuse; (3) possible current depression (Beck Depression Inventory-II, BDI-II>7) or intake of anti-depressants; and (4) possible dementia (Montreal Cognitive Assessment, MoCA<21/30) or intake of anti-dementia drugs.

All patients with PD were assessed on their regular anti-Parkinsonian drugs, including levodopa (*N* = 48), pramipexole (*N* = 25), selegiline (*N* = 16), piribedil (*N* = 13), amantadine (*N* = 8), entacapone (*N* = 4), and rasagiline (*N* = 1). The levodopa equivalent daily dose was calculated using the equation of Tomlinson et al. [15]. The severity of motor and non-motor symptoms was evaluated with the Movement Disorder Society-sponsored revision of the Unified Parkinson's Disease Rating Scale (MDS-UPDRS) Part III and I subscales, respectively.  Table 1 shows demographic and clinical features and neuropsychological measures.

### 2.2. Two Control Groups

We included two control groups: 88 age- and education-matched healthy controls (HC) from local communities and 40 matched patients with AD from the DementiaBank database [16].

For the HC group, exclusion criteria were (1) a history of significant neurological or psychiatric disorders; (2) alcohol or drug abuse; (3) possible current depression; and (4) possible dementia or mild cognitive impairment (MoCA<26/30). They completed the same assessments for cognition, mood, and sleep as patients with PD.

The DementiaBank database has 139 dementia patients assessed at the University of Pittsburgh School of Medicine. We only included AD patients matched with the other two groups in sex and age (20 women, age range 50-70 years, and mean age 62.2 years). We excluded patients diagnosed with other types of dementia, including mild cognitive impairment (*N* = 17), vascular diseases (*N* = 4), and other memory problems (*N* = 3).

### 2.3. Standard and Graph Theoretical Analyses

All participants completed a standard animal fluency test. For the PD and HC groups, we recorded and transcripted their verbal responses. For the AD group, we received their audios and transcripts from the database.

For the standard analysis, we defined five parameters: (1) the number of correct words without repetitions: all types of animals were accepted, including humans, insects, and mythical creatures (e.g., dragon); (2) the number of repetitions; (3) the number of incorrect words; (4) metalinguistic reference: the number of times participants talked about their responses (e.g., “did I say horses?”); (5) metacognitive reference: the number of times participants talked about their memory (e.g., “I really cannot think of any.”) or asked about the time (e.g., “how much time is left?”).

For the graph theoretical analysis, we transformed participants' verbal responses into directed speech graphs with Speechgraphs [12, 13]. In each directed speech graph, a node represented a word, and an arc represented the temporal link between an ordered pair of words (Figure 1(a)). We computed three graph parameters, including the density, diameter, and average shortest path length. The graph density is the ratio of arcs to the maximum possible number of arcs. The graph geodesic is the shortest path between two nodes (Figure 1(b)). The length of the maximum graph geodesic is the graph diameter. The mean length of all graph geodesics is the average shortest path length, also known as the characteristic path length of the graph.

### 2.4. Statistical Analysis

Data were analyzed with IBM SPSS Statistics 20. First, we examined group differences in the standard and graph parameters using one-way ANOVAs (two-tailed, *p* < 0.006 Bonferroni correction for eight tests). The ANOVA had a factor group (HC, PD, and AD) and a covariate age. Significant group differences were followed by pairwise comparisons (with Bonferroni correction).

Second, in PD, we examined whether the severity of motor or non-motor symptoms (MDS-UPDRS Part III or I subscores) correlated with the graph parameters that showed group differences using linear regression models (stepwise, *p* < 0.025 Bonferroni correction for two models).

## 3. Results

### 3.1. Group Differences in Standard Parameters

**Figure 2:**
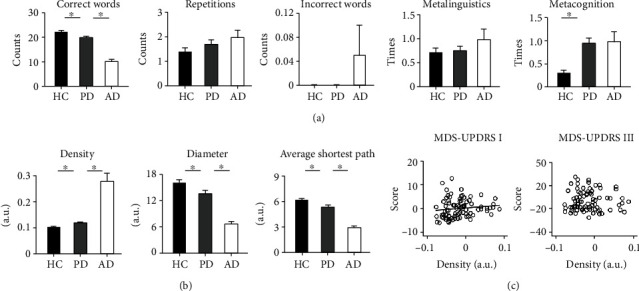
(a) Means and standard errors of correct words, repetitions, incorrect words, metalinguistic reference, and metacognitive reference in healthy controls (HC), patients with Parkinson's disease (PD), and patients with Alzheimer's disease (AD). (b) Means and standard errors of graph density, diameter, and average shortest path in each group. The asterisks (∗) indicate significant differences between PD patients and two control groups in standard and graph parameters. (c) In patients with PD, the density of speech graphs was correlated with the severity of non-motor symptoms (MDS-UPDRS I score) but not the severity of motor symptoms (MDS-UPDRS III score). Values were demeaned.

Figure 2(a) shows standard parameters in each group. Group differences were found in the number of correct words (*F*(2, 210) = 66.36, *p* < 0.001, and *η*_*p*_^2^ = 0.39) and metacognitive reference (*F*(2, 210) = 12.37, *p* < 0.001, and *η*_*p*_^2^ = 0.11), but not in the number of repetitions (*F*(2, 210) = 1.79, *p* = 0.169, *η*_*p*_^2^ = 0.02), number of incorrect words (*F*(2, 210) = 2.03, *p* = 0.134, and *η*_*p*_^2^ = 0.02), or metalinguistic reference (*F* < 1). The PD group generated fewer correct and non-repetitive words than the HC group (*p* = 0.008) but more correct and non-repetitive words than the AD group (*p* < 0.001). The PD group talked about their memory and time remaining more than the HC group (*p* < 0.001). Only the AD group generated incorrect words.

### 3.2. Group Differences in Graph Parameters

Figure 2(b) shows graph parameters in each group. Group differences were found in the density (*F*(2, 210) = 51.54, *p* < 0.001, and *η*_*p*_^2^ = 0.33), diameter (*F*(2, 210) = 38.40, *p* < 0.001, and *η*_*p*_^2^ = 0.27), and average shortest path (*F*(2, 210) = 42.55, *p* < 0.001, and *η*_*p*_^2^ = 0.29). The PD group showed higher density (*p* = 0.003), shorter diameter (*p* = 0.008), and shorter average shortest path length than the HC group (*p* = 0.008). The PD group showed lower density (*p* < 0.001), longer diameter (*p* < 0.001), and longer average shortest path length than the AD group (*p* < 0.001). In other words, speech graphs of the PD group were smaller and denser than those of the HC group but larger and more sparse than those of the AD group.

### 3.3. Correlations between Clinical Features and Graph Parameters in PD

Figure 2(c) shows correlations between graph parameters and clinical features in PD. The stepwise regression model for the MDS-UPDRS Part I subscore (*F*(1, 83) = 7.80, *p* = 0.006, and *R*^2^ = 0.09) included density (beta = 29.11, *t* = 2.79, and *p* = 0.006) but removed the diameter (beta = −0.14, *t* = −0.91, and *p* = 0.37) and average shortest path (beta = −0.11, *t* = −0.68, and *p* = 0.50). PD patients with more severe non-motor symptoms tended to produce smaller and denser speech graphs.

Linear regression model did not survive at the corrected threshold for the MDS-UPDRS Part III subscore (*F*(1, 84) = 4.24 and *p* = 0.043).

## 4. Discussion

In this study, we revisited the semantic disfluency in non-demented patients with PD. We replicated previous findings that patients with PD generated fewer correct and non-repetitive words than healthy controls [17–19] but more than patients with AD [20, 21]. More importantly, we examined the topology of participants' speech graphs and found that patients with PD produced smaller and denser speech graphs than healthy controls but larger and more sparse speech graphs than patients with AD. To be specific, the speech graphs of PD patients showed higher density, shorter diameter, and shorter average shortest path than those of healthy controls but lower density, longer diameter, and longer average shortest path length than those of AD patients. In PD, in addition, the density of speech graphs correlated with the severity of non-motor symptoms. PD patients who produced smaller and denser speech graphs exhibited more severe non-motor symptoms in daily living.

This study suggests that the graph theoretical analysis is more sensitive than the standard analysis to PD's problems in verbal fluency. For example, both approaches measured the repetition, but only the measures of the graph theoretical analysis (e.g., density, diameter, and average shortest path) showed significant group differences between PD patients and healthy controls. The repetition might reflect the impaired selection and programming processes of semantic fluency, which is associated with the left inferior frontal gyrus (LIFG) and basal ganglia.

Verbal fluency tasks involve several cognitive processes: (a) attention to search words from an abundant semantic store, (b) selection of appropriate words to produce, (c) programming of speech production, and (d) keeping track of the words that have already been produced to avoid repetitions. The dual stream model for language processing is a widely accepted model that describes two large-scale streams underlying different speech tasks [22]. The ventral stream is comprised of bilaterally superior and middle portions of the temporal lobes with a weak left-hemisphere bias, which supports the processing of sound-to-meaning information and is essential for auditory comprehension and semantic retrieval. The dorsal stream is comprised of the left posterior frontal lobe with a dominant position and left posterior temporal lobe and left parietal operculum, which supports the processing of sound-to-articulation (phonemic) information and is essential for speech learning and development. It is assumed that semantic and phonemic fluency is mediated by ventral and dorsal streams, respectively. The link between the phonemic fluency and the dorsal stream has been shown in a diffusion tensor imaging study with a large sample of de novo patients with PD [23]. Future studies are needed to examine whether damage to the ventral stream might have an impact on semantic fluency in patients with PD.

A selection mechanism will be applied when multiple verbal responses meet the instruction and compete for production. Previous studies showed that the LIFG is critical for this process. Thompson-Schill et al. (1997) used functional magnetic resonance imaging and found that the selection of information among competing alternatives in semantic tasks is associated with LIFG activity in healthy adults [24]. Robinson et al. (2010) also reported that generation of sentences was only impaired when selection is required in patients with LIFG lesions [25]. In addition, it has been suggested the basal ganglia support speech production through their role in programming and initiation by modulating the activity of premotor areas (supplementary motor area, presupplementary motor area, and dorsolateral prefrontal cortex) [26]. The disruption of striatal dopaminergic transmission in patients with PD may impair this modulation: The dopamine transporter availability in basal ganglia was directly associated with frontal functions (i.e., attention/working memory and executive functions) [27]. The decline of verbal fluency after pallidotomy in patients with PD may be due to surgical microlesions affecting cortical-basal ganglionic circuits involved in word generation processes [28, 29].

Semantic fluency relies on working memory and executive function, in addition to semantic knowledge. It has been described that the working memory deficits and executive dysfunction in patients with PD may result in semantic disfluency [30]. The impairments in working memory might result in the difficulty of keeping the search for new standard-compliant words and keeping track of produced words. The executive dysfunction leads to the deficits in selecting and producing appropriate words and inhibiting inappropriate words (i.e., repetitions and incorrect words). On the other hand, the difficulty of self-shifting may result in semantic disfluency. For example, Henry and Crawford (2004) [31] showed that PD patients shift from one semantic category to another with more effort than healthy adults.

Semantic disfluency has been linked to non-motor symptoms in PD in previous studies. PD patients with more severe depression or sleep disturbances tended to show worse performance in the semantic fluency tests [17, 32, 33]. In contrast, there was no correlation between PD patients' scores of semantic fluency task and their disease durations, levodopa intakes, severities of rigidity or tremor, or Hoehn-Yahr stages [34]. From another perspective, the semantic task could distinguish patients with PD and AD. Although patients with PD were impaired in semantic fluency, they were not as severe as patients with AD. The AD patients not only generated fewer correct words, but also repeated the same word with a smaller interval (e.g., dog-cat-horse-dog), indicating that the subtle deficit could underlie the differential diagnosis. In addition, the presence of semantic fluency deficits in PD has been identified as a potent risk factor of the development of PD related dementia [35].

This study has limitations. First, cultural and linguistic differences might be confounding factors. The AD group was from the database and was assessed by different experimenters and in different cultures. Some studies also showed that language and cultural differences have an impact on verbal fluency scores [36]; nevertheless, previous studies analyze the semantic data from different cultures (Greece and France) by using extended and unified methods [37]. It is worthy to examine the difference of semantic fluency in PD patients under different cultural backgrounds. Second, group differences and individual variability in graph parameters have not been linked with the structural integrity of brains. Third, some studies have shown that the performance of verbal fluency was improved when the PD patients were assessed with versus without levodopa treatment [38]. The PD patients scored higher in the semantic fluency task when they were treated with rasagiline than placebo [39]. It would be of interest to assess the impact of medication on the topology of speech graphs in future studies.

## 5. Conclusion

In this study, we analyzed the topology of speech graphs generated in a semantic fluency test. The speech graphs of patients with PD were smaller and denser than those of healthy controls but larger and more sparse than those of patients with AD. Moreover, PD patients who produced smaller and denser speech graphs exhibited more severe non-motor symptoms.

## Figures and Tables

**Table 1:** Demographic and clinical features, and neuropsychological measures of PD patients and healthy controls (means, standard deviations, and group differences).

Features/measures	PD patients (*N* = 86)	Healthy controls (*N* = 88)	Group differences (*p* values)
Female: Male	44 : 42	46 : 42	0.884
Age (years)	59.0 (9.5)	58.1 (7.0)	0.484
Education (years)	12.4 (3.2)	12.9 (2.4)	0.204
Montreal cognitive assessment	25.6 (2.4)	27.9 (1.4)	<0.001∗
Levodopa equivalent daily dose (mg)	243.3 (248.6)	—	—
*Motor symptoms*			
Hoehn and Yahr scale	1.4 (0.5)	—	—
MDS-UPDRS III: Motor examination	21.8 (12.6)	—	—
Disease duration (years)	1.6 (2.2)	—	—
Duration of motor symptoms (years)	2.8 (2.4)	—	—
*Other non-motor functions*			
MDS-UPDRS I: Non-motor experiences of daily living	5.3 (4.0)	—	—
Beck depression inventory-II	2.7 (2.0)	2.1 (1.7)	0.039
REM sleep behavior disorder screening questionnaire	3.7 (2.0)	1.9 (1.8)	<0.001∗
Epworth sleep scale	3.1 (3.2)	3.2 (2.3)	0.820

Note: MDS-UPDRS, the Movement Disorder Society-sponsored revision of the Unified Parkinson's Disease Rating Scale; group differences, *p* values of two-sample *t*-tests, or Chi-square test as appropriate; asterisks (∗), a significant difference (two-tailed, *p* < 0.007 Bonferroni correction for seven tests).

## Data Availability

Data have been uploaded to the figshare database https://figshare.com/articles/dataset/XW_data_2017-2019_xls/18393671.
